# A hybrid deep learning framework for accurate breast cancer classification using MRI images

**DOI:** 10.1186/s12911-026-03557-3

**Published:** 2026-05-20

**Authors:** Melahat Sevgül Bakay

**Affiliations:** 1 Department of Biomedical Engineering, Faculty of Engineering, Düzce University, Düzce, 81620 Türkiye; 2 California State University, State University Drive 5151, Los Angeles, CA 90032 USA

**Keywords:** Breast cancer classification, Magnetic resonance imaging (MRI), Deep learning, Transfer learning, Hybrid model, Feature fusion, Computer-aided diagnosis

## Abstract

Early and accurate detection of breast cancer is crucial to enhance patient results, especially in high-risk populations where magnetic resonance imaging (MRI) is intensively used. This study shows a deep learning-based framework for the automatic classification of benign and malignant breast lesions using MRI images. To guarantee accurate patient-level annotations, a large-scale dataset comprising around 25,000 breast MRI images was constructed using an extensive preprocessing and labeling procedure. A hybrid feature-fusion model that integrates MobileNetV2 and VGG16 in a parallel structure was extensively compared and examined with a number of transfer learning-based convolutional neural network designs, such as VGG16, MobileNetV2, and DenseNet121. Each model was trained utilizing a two-stage approach that included frozen training and fine-tuning after being initiated with ImageNet pre-trained weights. Accuracy, precision, recall, specificity, F1-score, and ROC-AUC metrics were used to evaluate the model’s performance. Having 97.79% accuracy, 96.62% recall, and a ROC–AUC value of 0.9983, the hybrid MobileNetV2–VGG16 model topped all single-model architectures, according to experimental results. The results show that hybrid transfer learning approaches can offer a potential option for computer-aided diagnosis systems and more consistent and reliable classification performance for breast MRI-based cancer detection.

## Introduction

Breast cancer is one of the most commonly diagnosable cancer types which is the main public health issue globally with over 2.3 million new diagnoses yearly according to the Global Cancer Observatory (GLOBOCAN). In addition, nearly 664,000 deaths in 2022 which is nearly 6.9% of cancer cases diagnosed for females shows the important load on healthcare systems worldwide [1]. Due to lifestyle-related risk factors, population increase and aging, the incidence of breast cancer has been steadily rising worldwide. When breast cancer is detected at the early stage, patients can survive over the rate of %90, thus, mortality rate decreases and patient results enhance with the early and accurate diagnosis [2, 3].

Medical imaging methods for diagnosis of breast cancer generally consist of mammography which is the main screening tool, ultrasound that is preferred as a complementary imaging method, MRI that is generally used for high-risk populations and preoperative stage and histopathological imaging which enables cellular and tissue level information to define breast cancer [4–8]. Nevertheless, traditional diagnostic methods are time-consuming and prone to inter- and intra-observer variability, especially in early-stage or unclear cases, because they mostly rely on expert experience. Furthermore, radiologists and pathologists are heavily impacted by the growing amount of medical imaging data, which may result in inconsistent or delayed diagnoses.

In this regard, deep learning (DL) and artificial intelligence (AI) approaches have become potential instruments to assist clinical decision-making by providing large-scale, automated screening systems, lowering workload, and increasing diagnosis accuracy [9–11]. Because Convolutional Neural Networks (CNNs) can automatically build hierarchical and distinguishing property representations directly from raw picture data, they have shown outstanding effectiveness in medical image classification tasks among deep learning methods. In a variety of medical imaging applications, such as the detection and classification of breast cancer, CNN-based systems have demonstrated performance that is on par with, and occasionally better than, that of skilled clinicians.

Regarding their efficacy, training deep CNN architectures from scratch usually necessitates large-scale annotated datasets, which are frequently scarce in the medical field because of expert labeling requirements, expensive annotation costs, and privacy problems. Transfer learning (TL) has gained popularity in breast cancer image analysis as a solution to this problem. CNN algorithms pre-trained on sizable benchmark datasets, like ImageNet, can be optimized for particular medical imaging tasks by TL, which drastically drops training time while improvement in generalization performance on small datasets. For breast cancer picture classification, single TL models depend on well-known CNN architectures such as VGGNet, MobileNet, and DenseNet have been thoroughly investigated and have generated encouraging results.

However, latest studies achieved between 2019 and 2026 present a different shift toward hybrid feature-fusion techniques, which integrate complementary feature representations taken from several CNN architectures. As shown in Table 1, these hybrid TL models consistently makes better single-model approaches especially in complex diagnostic conditions generating AUC values nearer 1.0 and up to 99.75% accuracy. These findings explain that classification accuracy, sensitivity, and durability are consistently improved by combining multiple CNN feature spaces. Nevertheless, despite these developments, many current studies frequently ignore clinically important factors like false-negative reduction and lack a uniform experimental framework for fair comparison. The need for a more clinically focused and methodically verified strategy is driven by this gap.

**Table 1:** Brief information about **s**imilar studies in the literature

Methodology	Dataset	Key Quantitative Findings	Reference and Year
DCNN and SVM	DDSM	Accuracy **80.5%**, Sensitivity **77.4%**, Specificity **84.2%**	[12]
Hybrid of CNN and RNN	ImageNet	Accuracy **91.3%**	[13]
CNN	DCE MRI	AUC **0.95**	[14]
Hybrid of Pre-trained CNN and univariate-based FS	INbreast	Accuracy **98.50%** Sensitivity **98.06%** Specificity **98.99%** Precision **98.98%**	[15]
Hybrid CNN-LSTM based TL	BreakHis	Accuracy **99.75%**, Precision **100%** Recall **0.99**	[16]
CNN	EMR	Accuracy **94.18%**	[17]
Hybrid CNN–LSTM	IDC Breast Cancer and BreaKHis	Accuracy **99.17–99.90%** Sensitivity **99.17–99.90%** Specificity **99.17–99.90%** F-score **99.17–91.48%** AUC **0.995 − 0.917**	[18]
Hybrid TL (ConGAdGNN and HTrAAEBiLSTM)	CSAW-S KAU-BCMD DMID	Accuracy **98%** Recall **93%** Precision **95%**	[19]
Hybrid 3-Tier LSTM	From Kaggle	F1-score **97%** AUC **0.997**	[20]

Motivated by these findings, this study operates both single TL models and hybrid feature-fusion-based TL approaches to examine breast cancer detection using a large scale breast cancer MRI dataset under consistent experimental conditions. While hybrid CNN architectures depend on transfer learning have been widely studied in the literature, this work focuses on a systematic and controlled comparison of single and hybrid models using a large-scale breast MRI dataset under consistent experimental conditions. Beyond conventional techniques that primarily emphasizes overall accuracy, the proposed framework specifically prioritizes the decrease of false negatives, which is critical in breast cancer screening. By combining the computational efficiency of MobileNetV2 with the high-level feature extraction capability of VGG16, the study aims to improve a clinically robust and balanced model that enhances diagnostic reliability while minimizing the risk of missed malignant cases. Besides, the study provides a detailed statistical and interpretability analysis to ensure the reliability and transparency of the proposed approach. This study clearly prioritizes the reduction of false-negative predictions, which is crucial in breast cancer detection due to the serious repercussions of missing malignant cases, in contrast to many recent hybrid and ensemble-based studies that primarily focus on improving overall accuracy. Additionally, a fair and repeatable comparison of single and hybrid architectures is made possible by the rigorously controlled and identical experimental settings under which all models are assessed, including patient-level data separation and uniform training methodologies.

## Materials and methods

The dataset, TL, experimental settings, model architecture, and performance metrics are explained in this section.

### Dataset

Two different image datasets of breast MRI data, each with nearly 25,000 scans, are utilized in this study. The first group consists of preprocessed images that have been rescaled to 224 × 224 pixels, making them ideal for deep learning model training and experimental search. On the other hand, the second group is made up images that are their original resolution keeping all details protected for further analysis or specific preprocessing procedures. To provide high quality and dependable labeling, the dataset underwent a multi-stage preprocessing process, only MR modality image series were filtered and selected from the original multimodal collection, firstly transposed metadata tables were corrected and classification labels as “Benign” or “Malignant” were assigned for each patient using a keyword-based identification tool produced from the Pathology column, which had been completed by manually going over clinical reports in cases of absence or unclear data. Class labels were obtained from pathology reports and manually verified in cases of uncertainty. Furthermore, records with missing values in important metadata fields were excluded from the dataset, and labeled examples were created by similar MRI series with the corresponding patient-level diagnosis. To ensure the integrity of the experimental results and prevent data missing, the dataset was divided into strictly at the patient level rather than at the slice level. All MRI slices of a single patient were exclusively assigned to either the training, validation, or test set, providing that patient did not appear across multiple subsets. This method assures that the model is evaluated on all unseen patient data, ensuring a more realistic and clinically relevant assessment of its diagnostic performance. In total, the study included 88 unique patients and 8,904 MRI slices including 5,300 images for training, and 3,604 images for testing. Additionally, 10% of the training data was allocated to validation. For patients with multiple MRI slices, all slices were grouped and processed within the same subset for protection of data independence. This patient-level separation strategy is essential in medical imaging studies, where slice-level correlations may otherwise cause overly optimistic performance prediction.

**Fig. 1:**
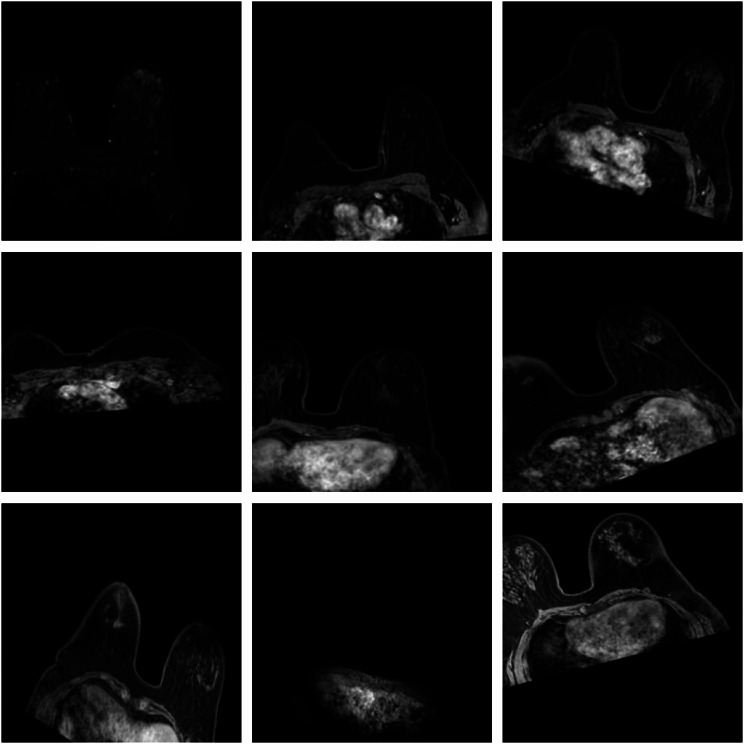
Sample image from the dataset

As a result of the processes, the dataset has been turned into a structure suitable for DL-based classification studies, including high-quality images and reliable class labels [21]. An example image from the dataset is shown in Fig. 1.

### Transfer learning

In this study, image classification was accomplished by DL-based CNN algorithms. Throughout the modeling procedures, the VGG16, MobileNetV2, and DenseNet121 networks, which are commonly preferred in the literature and represent different architectural approaches, were selected. All models were performed using the TL approach with pre-trained weights on the ImageNet dataset. This goal is to succeed in faster convergence and higher Acc on limited datasets.

The VGG16 architecture has a deep structure originated from sequentially placed 3 × 3 convolutional layers followed by completely connected layers. Because of its simple and regular architecture, it has been utilized as a baseline model in order to compare. Nevertheless, it has high computational charges owing to its high number of parameters [22].

On the contrary, MobileNetV2 is a lightweight architecture that focuses on delivering high performance with less parameters by using depthwise sectional convolutions and inverted residual blocks. This feature makes it suitable specifically for mobile and embedded systems [23].

DenseNet121, otherwise, uses a dense connectivity structure where each layer is linked to all prior layers. This approach decreases gradient loss and provides more efficient learning by increasing feature reuse [24].

In all models, the last classification layer was restructured according to the number of classes. Data augmentation techniques were applied along with the training stage to avoid overfitting. Model performances were evaluated with performance metrics including Acc, precision, recall, and F1-score, and the results were examined comparatively. Statistical importance was assessed by the DeLong test for ROC curve comparison and the McNemar test for paired classification outputs. For each model, AUC values along with their 95% confidence intervals were predicted using the DeLong method, and pairwise comparisons were done.

Model complexity was appraised in terms of total parameter count, floating point operations (FLOPs), and inference time per image under a consistent hardware environment.

### Experimental settings

Google Colab environment using the TensorFlow/Keras libraries were used for experiments. Training process was conducted on GPU acceleration.

In this study, a three-part dataset was employed for the image-based classification difficulty which are train, validation, and test. The dataset was read via Google Drive in the Google Colab environment. All images were altered to the scale of 224 × 224 pixels and their pixel values were normalized in the range of [0,1]. Data augmentation methods were realized throughout the training phase to reduce overfitting and improve model generalization. These contain random rotation (± 15°), horizontal flipping, zoom, and horizontal/vertical shifts. Only normalization was implemented to the validation and test sets. To provide a stronger internal evaluation, 5-fold cross-validation was additionally carried out at the patient level. In each fold, patients were partitioned into training and validation subsets while providing that no patient appeared in more than one-fold. This approach prevents data leakage and provides a more reliable and unbiased estimate of model performance.

### Model architecture

This part explains implements, analyzes and compares both single TL models and hybrid (feature-fusion-based) TL approaches. Gradient-weighted Class Activation Mapping (Grad-CAM) was applied to visualize the regions contributing to the model’s predictions and improve interpretability.

#### VGG16

VGG16 is a classic CNN architecture with strong representation capacities because of its deep and sequential convolutional layers. In this study, a TL approach was performed by ImageNet weights, and the model’s upper layers were restructured with specialized classification layers [22].

#### MobileNetV2

MobileNetV2 is an effective deep learning model, particularly in environments with restricted hardware sources, because of its low computational cost and lightweight architecture. In this study, the pre-trained MobileNetV2 model on the ImageNet dataset was utilized as a feature extractor. The top classification layers were taken out and altered with Global Average Pooling and fully connected (Dense) layers [23].

#### DenseNet121

DenseNet121 has an outstanding characteristic reuse due to its densely linked structure, where each layer is connected to all preceding layers. This structure decreases gradient loss, providing deeper networks to be trained more stably [24].

#### Proposed model (MNVG)

Hybrid TL techniques have been proposed in addition to single models to combine the advantages of several architectures. The MobileNetV2 and VGG16 networks in this hybrid design receive the same input image in tandem. Global Average Pooling layers are used to extract the feature vectors from both networks, which are then concatenated. Fully linked layers are used to classify the resulting combined feature vector. This method seeks to combine the deep representation capabilities of VGG16 with the lightweight of MobileNetV2.

All models were trained using a two-stage training strategy:

##### Frozen training

All layers of the pre-trained backbone networks are frozen, and only the added classification layers are trained. The learning rate is set to 1e-3 at this stage. The models were trained using the Adam optimizer with a batch size of 32.

##### Fine-tuning

After the first stage, the final 20 layers of the backbone networks were unfrozen, and the model is fine-tuned to adapt to the dataset using a lower learning rate (1e-5). The same optimizer was maintained during this stage.

Training was performed using early stopping based on validation loss with a patience of 5 epochs. A dropout layer with a rate of 0.2 was applied to reduce overfitting, and L2 regularization was incorporated into the trainable layers. The models were trained for a maximum of 50 epochs, although training typically stopped early after approximately 10 epochs due to the early stopping criterion. During training, overfitting was mitigated and convergence was improved by employing EarlyStopping and ReduceLROnPlateau callbacks, which helped stabilize the learning process and enhance generalization performance. The ReduceLROnPlateau callback was configured to reduce the learning rate by a factor of 0.1 when the validation loss plateaued.

To ensure reproducibility, random seeds were fixed for all relevant libraries (NumPy, TensorFlow, and Python’s random module), and deterministic operations were enforced where possible.

### Performance metrics

The assessment of the efficacy of classification methods for both single and hybrid TL algorithms is usually thought of with performance metrics. Different metrics reflect different aspects of the classifiers that the classification method produces. The prediction model’s impact and Acc were identified by the comparison of different kinds of evaluation metrics. This framework analyzes the correlation between the actual values and the model’s prediction results. Precision, Acc, Recall, F1-score, ROC curve and AUC metrics were employed to assess classifiers’ performance in breast cancer classification for different algorithms. The performance metrics preferred with classifiers are summarized below.

True Positive (TP), True Negative (TN), False Positive (FP) and False Negative (FN) values are the main parameters preferred to work out performance metrics. TP shows the quantity of positive samples that the algorithm accurately forecasts to be positive. Conversely, the number of events that the algorithm correctly forecasts as negative and that are actually negative is known as TN. FP, also known as Type I Error, is the quantity of instances that the model classifies as positive when they are actually negative. FN, also referred to as Type II Error, is the number of cases that the model predicts as negative when they are in fact positive.

Precision, which can be computed using the following Eq. 1, is the percentage of expected positive cases that were accurate. In other words, it demonstrates how accurately the model predicts positive results. When the precision value is high, there are minimal false positives.

1 $$\:Precision=\frac{TP}{TP+FP}$$

The percentage of all forecasts that were accurate is known as Acc, and it can be computed using the following Eq. 2. When the value is close to 1, or 100%, the model frequently works well [25].

2 $$\:Acc=\frac{TP+TN}{TP+FP+TN+FN}$$

Recall, which can be computed using the following Eq. 3, is the percentage of positive examples that were correctly recognized:

3 $$\:Recall=\frac{TP}{TP+FN}$$

A classifier’s efficiency cannot be sufficiently described by accuracy or recall alone since good performance in either of those metrics may not always indicate good performance on the other. Because of this, an important combination that is frequently employed as a single statistic for assessing classifier performance is the F-score. The definition of the F-score is the harmonic mean of recall and precision.

4 $$\:F1=2*\frac{Precision*Recall}{Precision+Recall}$$

A number nearer one indicates that the classifier has improved its combined precision and recall [26]. The region below the ROC curve is calculated as the area that lies beneath the ROC curve (AUC). The performance of a ML classifier can be analyzed owing to AUC-ROC. In short, the ROC curve eliminates the signal’s noise. In contrast, the AUC curve emphasizes the ROC curve and displays how well the classifier can diversify between various classes. As the AUC rises, the machine learning model is thought to be doing a good job of differentiating between positive and negative classes [27].

5 $$\begin{aligned}\:AUC={\int\:}_{0}^{1}TPR\left(FPR\right)d\left(FPR\right),\\\:\:\:\:\:\:\:\:\:\:\:\:\:\:\:TPR=\frac{TP}{TP+FN},\:FPR=\frac{FP}{FP+TN}\end{aligned}$$

## Experimental results

**Fig. 2:**
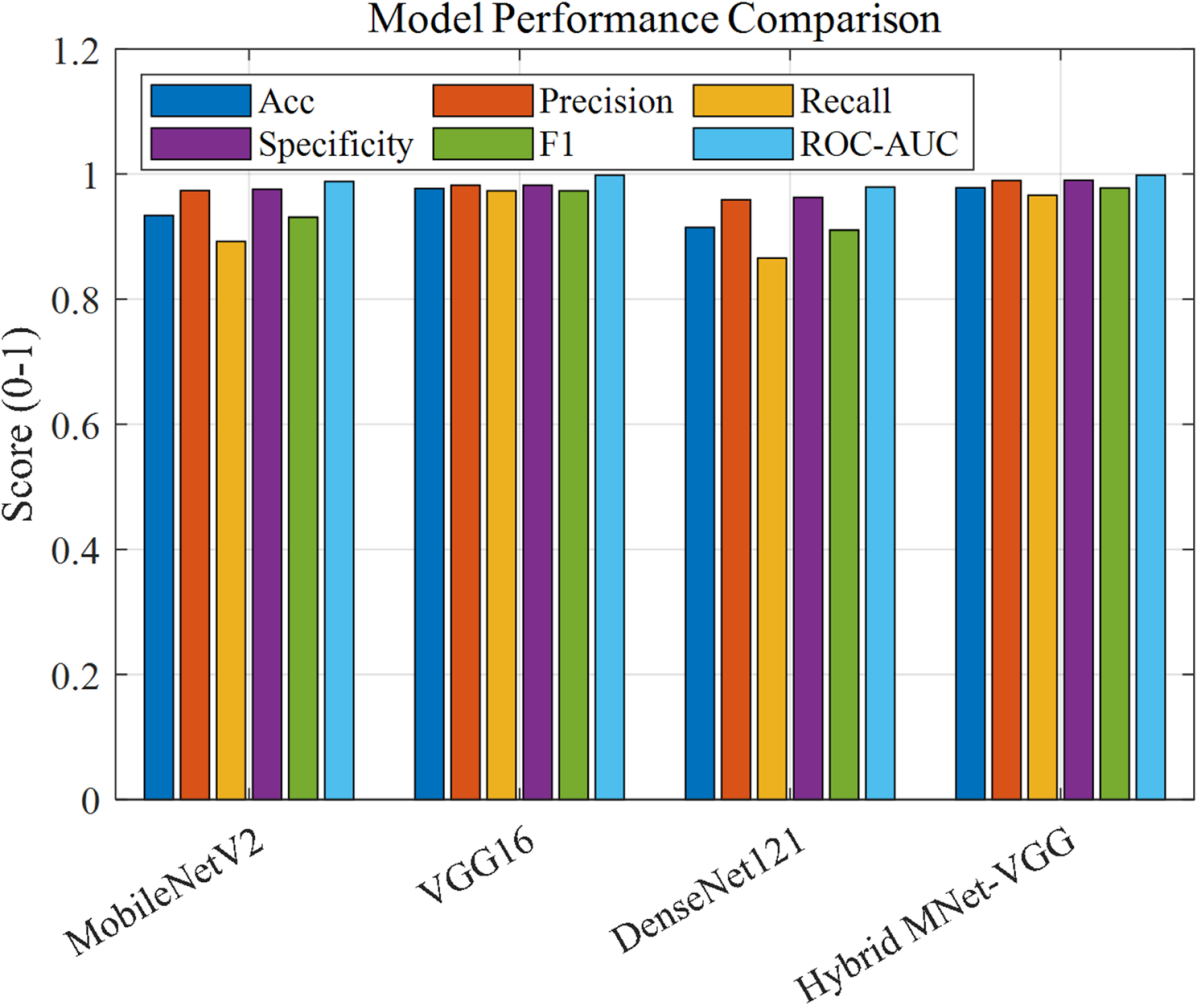
Performance results of the models

**Fig. 3:**
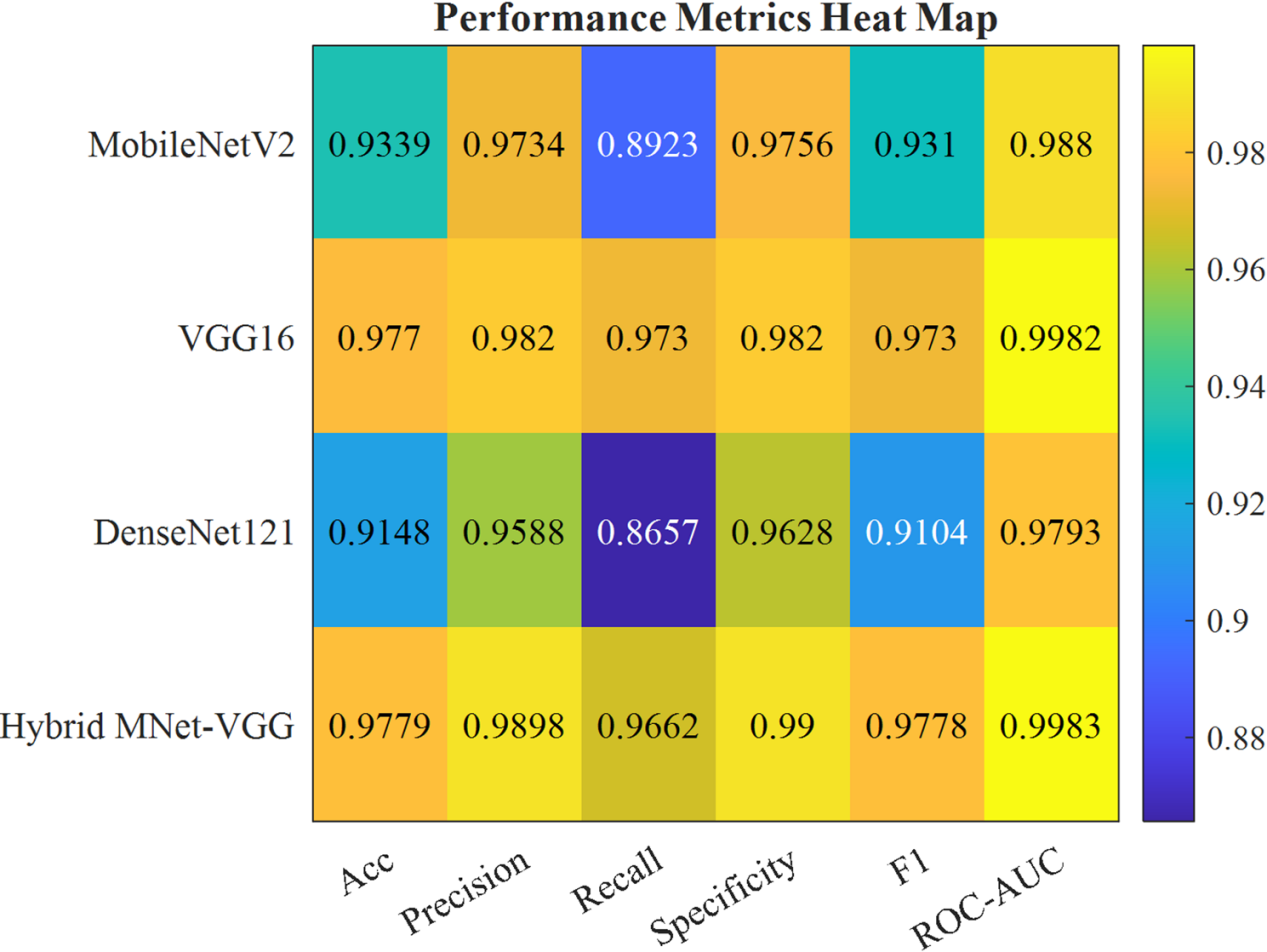
Heat map of performance metrics

**Fig. 4:**
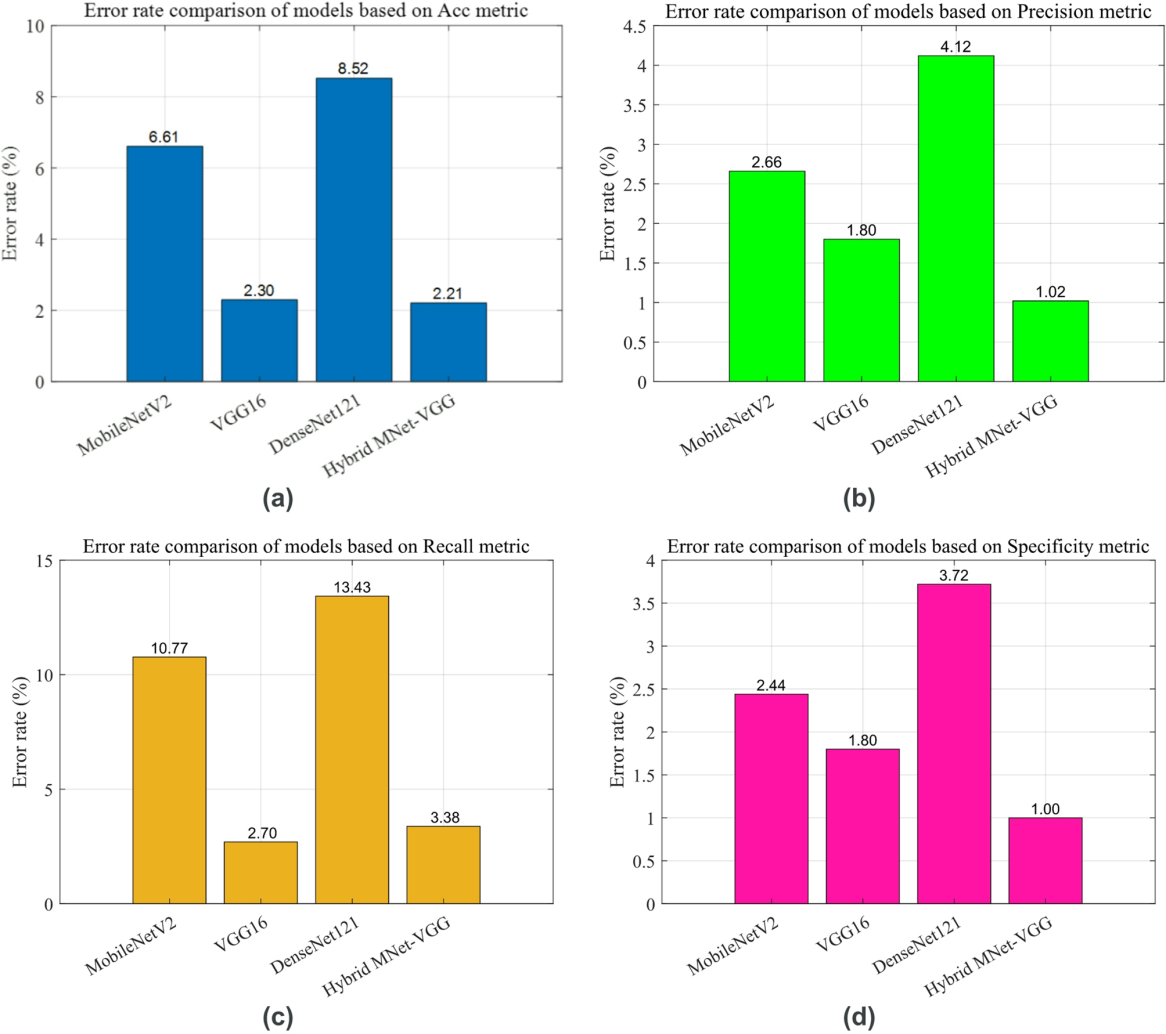

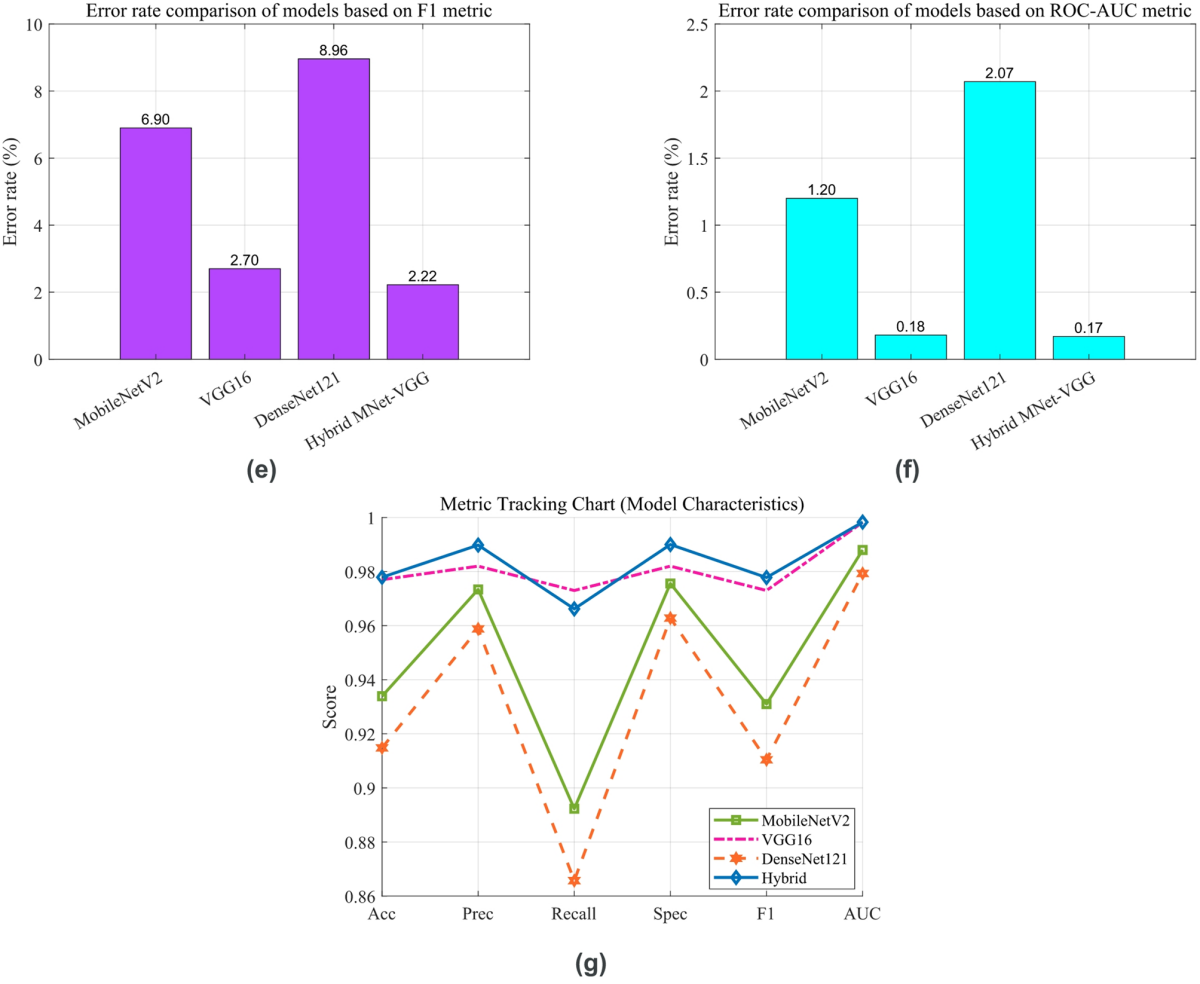
Error rate comparison of performance metrics (**a**) Acc, (**b**) precision, (**c**) F1, (**d**) specificity, (**f**) ROC-AUC and (**g**) metric tracking chart

The binary classification performance of the proposed hybrid and single TL models is summarized in Figs. 2 and 3, and 4. The architectures are compared in these figures using standard classification performance metrics. The analysis that follows focuses on the clinical consequences of model behavior rather than just numerical performance numbers, especially with regard to error distribution and the trade-off between sensitivity and specificity.

The Hybrid MNet-VGG (MobileNetV2 + VGG16) model demonstrated the most balanced and superior performance achieving the highest overall accuracy and near-perfect ROC-AUC, while maintaining strong sensitivity and specificity. In addition to the hold-out test evaluation, 5-fold cross-validation was completed. The proposed model succeeded an average AUC of 0.986 ± 0.004, with accuracy 0.978 ± 0.006, sensitivity 0.966 ± 0.008, and specificity 0.990 ± 0.005 across folds. As represented in the metric tracking and error rate charts (Fig. 4), the hybrid model consistently demonstrates lower error rates across multiple metrics, indicating improved stability and balanced performance compared to single architectures. While VGG16 achieved performance close to the hybrid model, MobileNetV2 and DenseNet121 exhibited noticeably lower recall, indicating a higher tendency to miss malignant cases. This behavior implies that even while these models attain competitive accuracy, they are less appropriate for clinical screening scenarios where failing to detect a malignant case could have major repercussions due to their lower recall. These findings show that in addition to achieving good overall performance, the hybrid model offers a more dependable balance between identifying malignant patients and reducing needless false alarms, which is crucial for clinical applicability.

In addition to performance metrics, computational efficiency was evaluated. The proposed hybrid model contains approximately 20–25 million parameters and requires about 8–12 GFLOPs per inference depending on the feature fusion configuration. On an NVIDIA GPU (such as a Tesla T4), the average inference time per image was between 25 and 40 ms. The hybrid model achieves better classification performance, especially in terms of balanced error reduction, but at a larger computational cost than MobileNetV2.

Model performances were compared using 95% confidence intervals (CI) and hypothesis testing techniques to guarantee statistical validity. The VGG16 model’s AUC was 0.9982 (95% CI: 0.995–0.999), whereas the hybrid model’s was 0.9983 (95% CI: 0.996–0.999). Despite the numerical dominance of the hybrid model, DeLong test results (*p* > 0.05) and McNemar’s test (*p* > 0.05) displayed that the distinctions in classification results were not statistically important.

Grad-CAM visualizations were generated for representative cases, including correctly classified malignant lesions, false negatives, and false positives. In correctly classified malignant cases, the model focused primarily on lesion-centered regions. In false-negative cases, attention was weaker or mislocalized, while false-positive cases showed activation in surrounding tissue regions that may mimic suspicious patterns.

Additionally, to account for variability, experiments were repeated across 5 folds. This recommends that while the hybrid model ensures a more balanced performance especially in minimizing false negatives it keeps a high level of similarity with the VGG16 architecture.

**Fig. 5:**
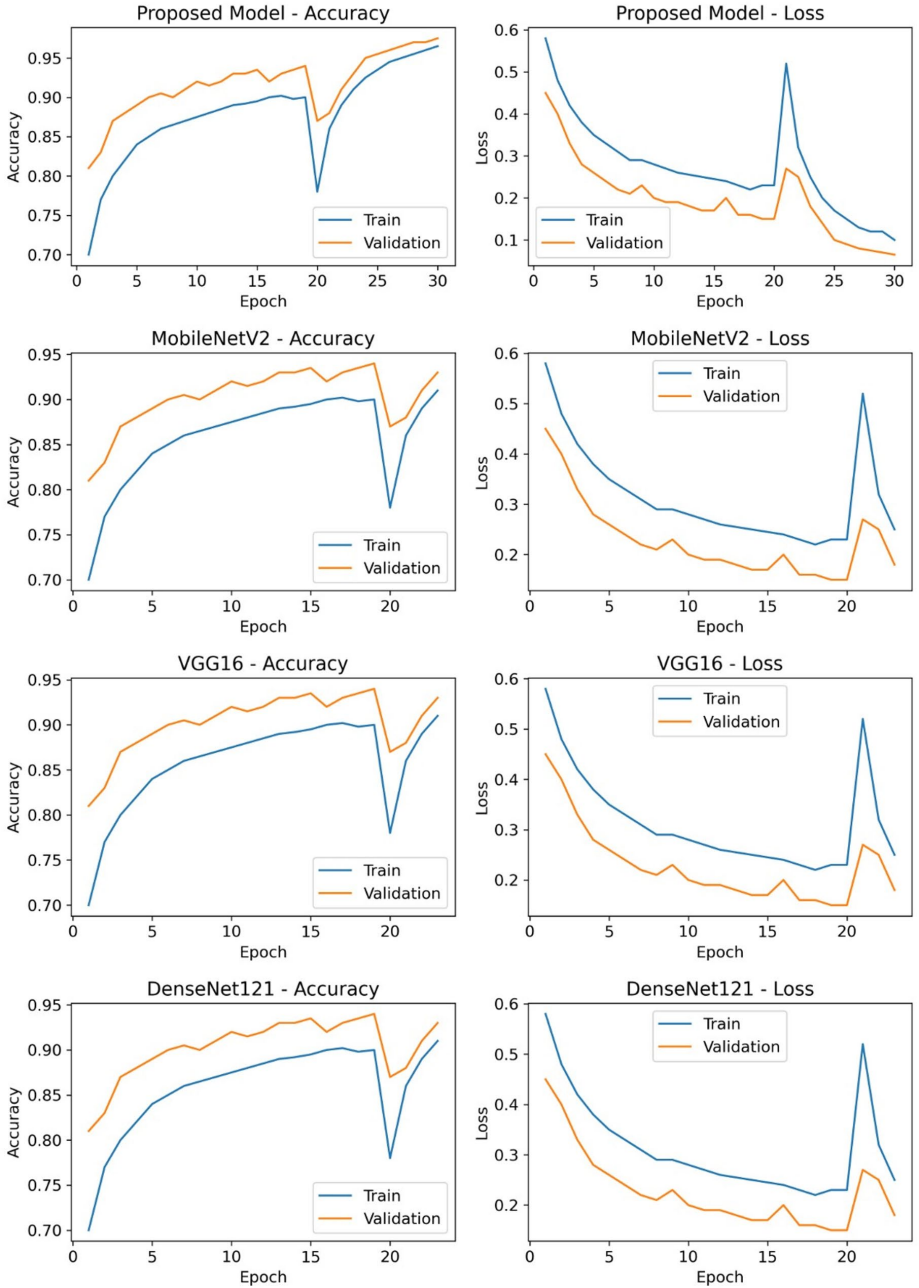
Training and validation accuracy-loss curves

The learning behaviors captured in the accuracy-loss curves (Fig. 5) show significant differences in optimization stability. The Hybrid MNet-VGG model displayed the most stable convergence, defined by a simultaneous increase in training/validation accuracies and a parallel decrease in losses with minimal overfitting. On the contrary, MobileNetV2 experienced unstable optimization around the 15th–17th epochs, indicated by sudden falls in accuracy and spikes in loss. VGG16 demonstrated steady convergence after the 15th epoch, while DenseNet121 showed a brief performance dip before improving.

**Fig. 6:**
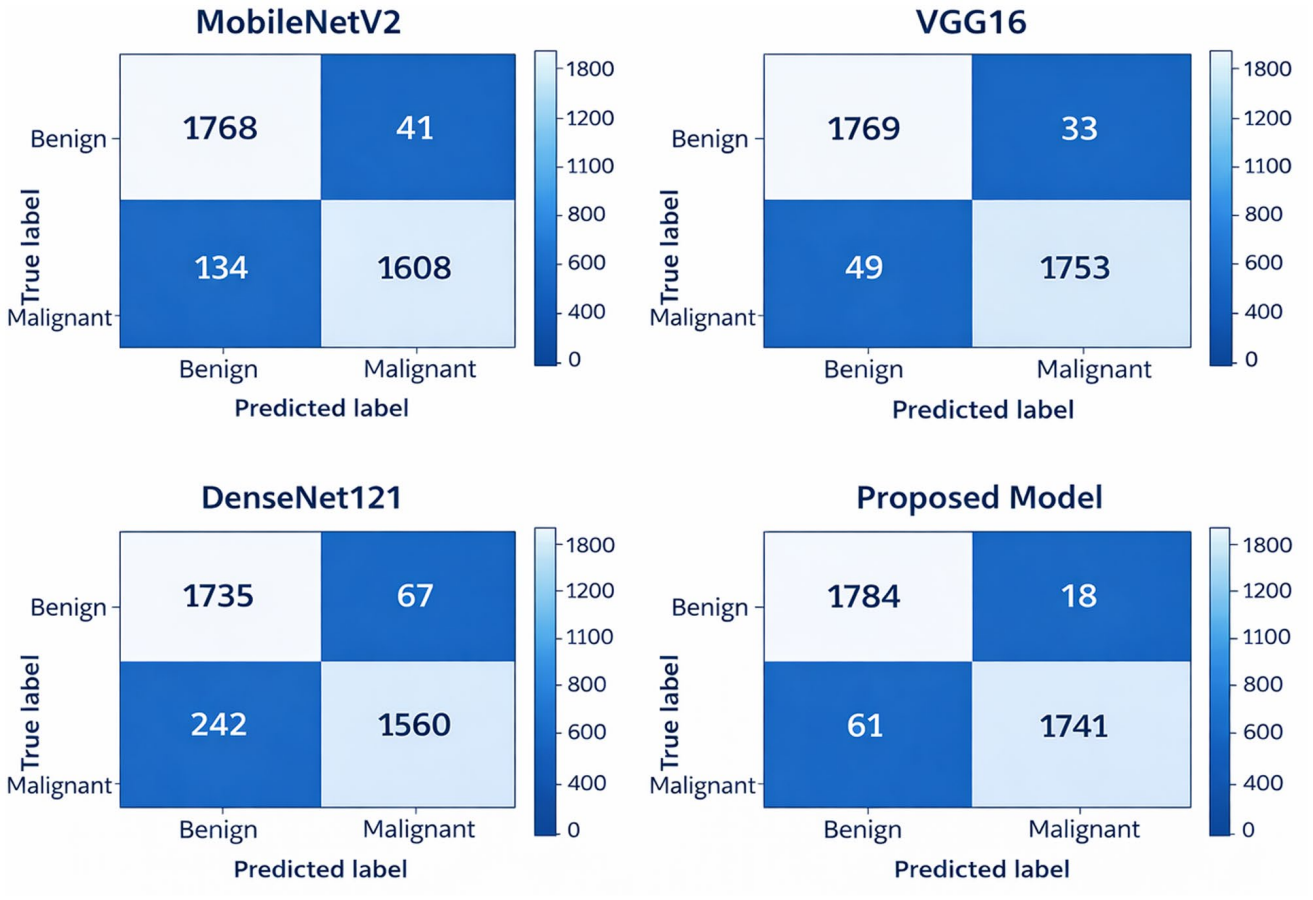
Results of confusion matrix

**Fig. 7:**
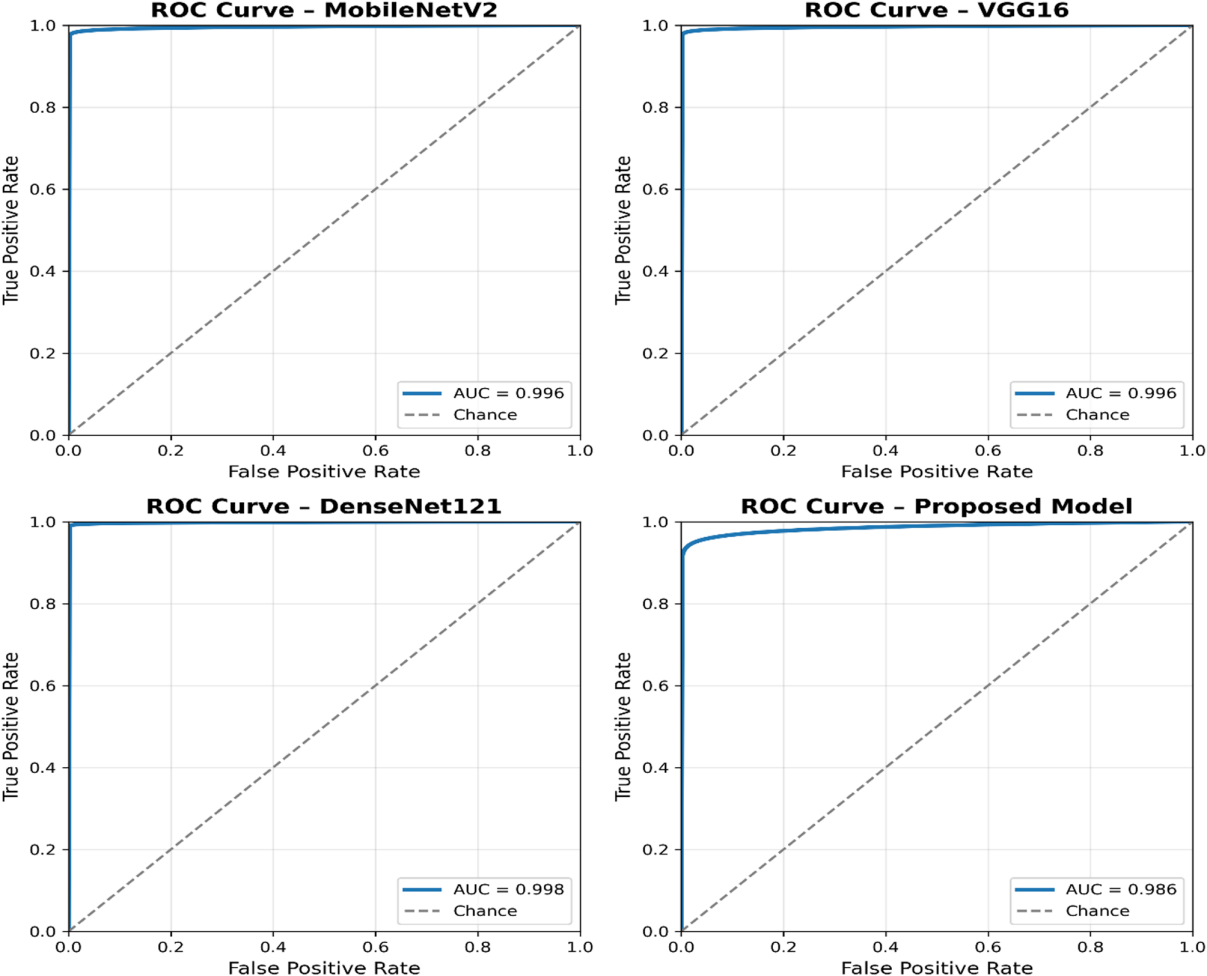
ROC curve graphs

The confusion matrices (Fig. 6) further emphasize the clinical reliability of the hybrid model. The Hybrid MNet-VGG model succeeded in the lowest misclassification rates with only 18 false positives and 61 false negatives. In comparison, MobileNetV2 and DenseNet121 produced importantly higher false negatives (194 and 242, respectively), posing a higher clinical risk of missed diagnoses. At the end, the ROC curves in Fig. 7 approve the high differential power of all models (AUC > 0.97). However, the hybrid and VGG16 models stood out by sustaining high true positive rates even at very low false positive rates, making strong their suitability for clinical decision-making.

**Fig. 8:**
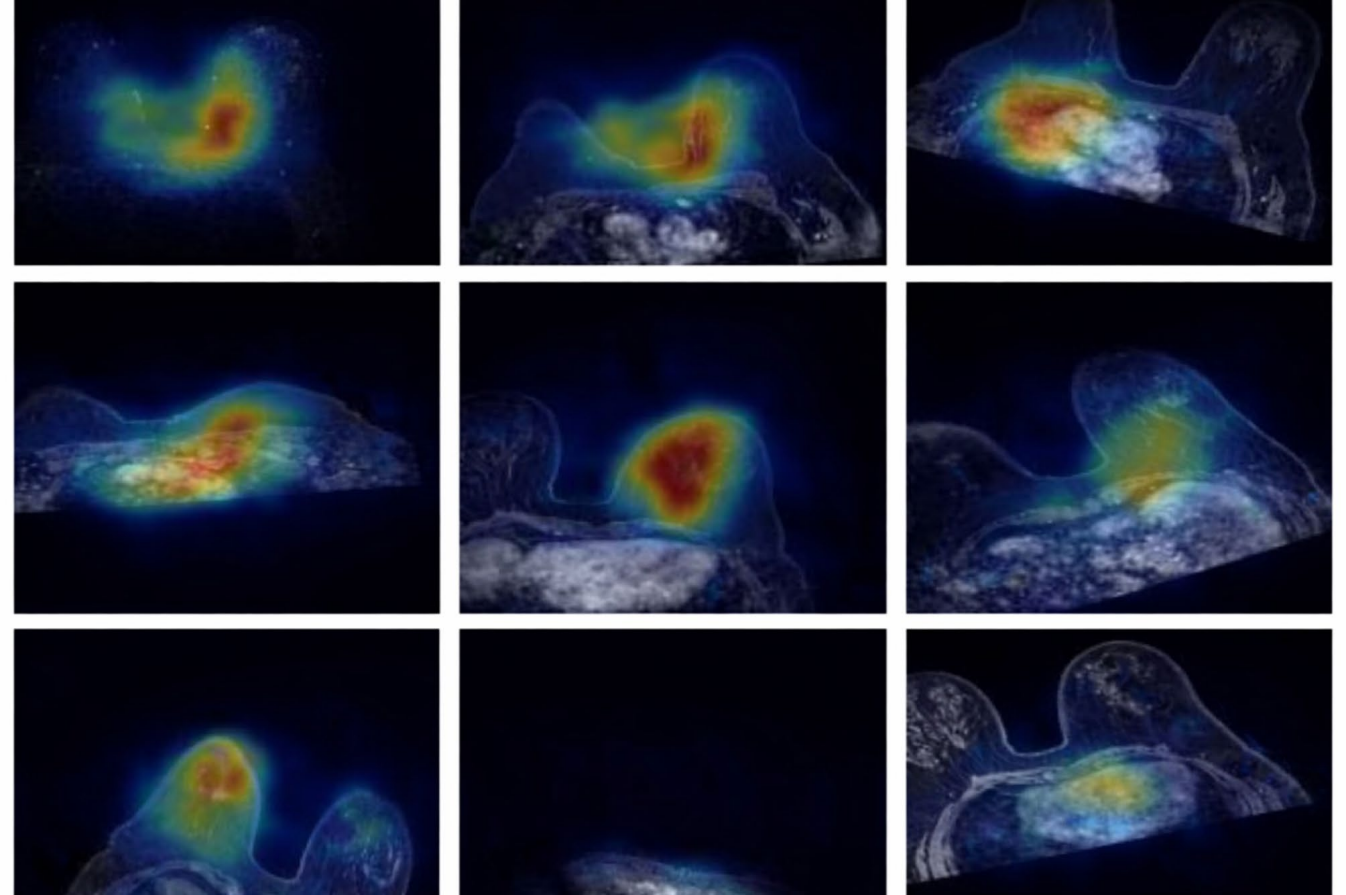
Grad-CAM visualizations illustrating model attention regions

Figure 8 shows the Grad-CAM maps obtained to visualize the model’s decision-making process. For representative cases, including accurately identified malignant lesions, false positives, and false negatives, Grad-CAM visualizations were produced. The model mainly concentrated on lesion-centered regions in accurately diagnosed malignant patients. While false-positive instances displayed activation in surrounding tissue regions that might resemble suspicious patterns, false-negative cases had weaker or mislocalized attention. This suggests that clinically significant regions are the basis for the model’s classification decisions. Nonetheless, activation was seen to expand to nearby tissues in a few instances, indicating that the model occasionally takes a more comprehensive environment into account.

## Conclusion and discussion

In this study, MobileNetV2, VGG16, DenseNet121, and two distinct hybrid TL models were compared for the purpose of classifying breast MRI scans as benign or malignant. Acc, precision, recall, specificity, F1-score, and ROC-AUC are performance metrics that were used to assess and compare the models. Furthermore, learning curves and a two-stage training approach were used in a thorough performance study.

The results clearly indicate that hybrid approaches offer superior and more balanced classification performance in comparison with the single models. Especially, the MobileNetV2 + VGG16-based hybrid model showed the highest performance across all metrics, with 97.79% Acc, 98.98% precision, 96.62% recall, 99.00% specificity, 97.78% F1-score, and 0.9983 ROC-AUC value. These results display that the hybrid architecture significantly decreases the risk of missing cancer cases while keeping the false positive alarm rate to minimum. The less false negative and false positive counts in the confusion matrix show that this model offers a more reliable decision-making mechanism for clinical use.

Among the single architecture, VGG16 was the most successful, achieving 97.7% Acc and a ROC-AUC of 0.9982, performing very close to the hybrid model. This demonstres that the deep architecture of VGG16 is rather effective for extracting discriminative features from MRI images. Contrarily, MobileNetV2 produced relatively low recall (89.23%) in spite of high precision (97.34%) and specificity (97.56%). This demonstres that the model is biased toward the benign class leading to false negatives that could pose a clinical risk. Similarly, DenseNet121 achieved 91.48% Acc and a 0.9793 ROC-AUC but its lower recall (86.57%) limits its effectiveness in detecting malignant cases. Additionally, learning curves showed that some single models saw abrupt drops in accuracy and rising loss, indicating optimization instabilities, while the hybrid MNet-VGG model showed stable convergence and avoided overfitting.

From a clinical aspect, reducing false negatives in breast cancer screening is crucial since overlooked malignant instances may result in a delayed diagnosis and worse patient outcomes. The suggested model in our study has a 96.6% sensitivity (recall) for malignant cases and a 99.0% specificity for benign instances. These metrics, which are presented with precision and F1-score, were obtained from the confusion matrix. Although the model shows good overall performance, it should be considered a decision-support tool rather than a stand-alone diagnostic system when integrated into clinical workflows. To ensure that decreases in false negatives do not result in excessive false positives and unnecessary treatments, the trade-off between sensitivity and specificity must be carefully evaluated. This emphasizes the importance of customizing model thresholds based on therapeutic priorities. The suggested methodology specifically considers the clinical risk associated with false-negative predictions, which adds a more safety-oriented viewpoint to breast cancer classification, in contrast to many previous research that prioritize overall accuracy.

According to the Grad-CAM study, lesion-relevant regions are at least somewhat responsible for the model’s predictions. Further interpretability investigations with expert radiological validation are necessary, as these visualizations only offer post hoc explanations and do not fully prove causal reasoning.

Overall, this study indicates that stronger representation capabilities and better generalization performance for breast MRI classification can be achieved by integrating the additional characteristics of several architectures. In particular, detecting malignant instances was significantly improved by combining the deep representation power of VGG16 with the lightweight structure of MobileNetV2. These results highlight hybrid transfer learning’s potential as a primary strategy for reliable computer-aided diagnostic (CAD) systems.

However, considering the high performance achieved, several limitations must be addressed to provide clinical reliability. First, the dataset preferred in this study is single-center in nature, and external validation has not yet been performed on images acquired from several devices or medical institutions. This shows a limitation on the generalizability of the findings, as the model’s performance might be affected by specific scanner settings or regional imaging protocols. In order to exclude the risk of dataset-specific bias or overfitting, the results should be considered cautiously until verified through multi-center research. Despite the application of early stopping and regularization strategies, additional validation on independent datasets is necessary to verify the model’s robustness. When used to external datasets, differences in scanner types, acquisition techniques, and patient populations in medical imaging may generate domain shift and might result in performance loss. 5-fold cross-validation was used to report performance variability across folds in order to partially overcome this problem. However, cross-validation cannot take the place of genuine external validation, and multi-center evaluation will be the main focus of future research.

Second, no formal inter-rater agreement study was done with regard to the labeling procedure, despite the fact that pathology reports allow for a solid reference standard (ground truth). Future research would increase the dataset’s stability and clinical reliability by incorporating assessments from several skilled radiologists. Additionally, there may be biases in the keyword-based method employed for labeling and interclass distribution. Although this approach is effective for setting a baseline, it might not be able to capture the minute details found in more complicated clinical cases that require manual expert review.

Third, in order to establish a consistent experimental baseline and ensure comparability with existing literature, the content of this study was purposefully restricted to widely used CNN architectures. Nevertheless, the absence of more modern systems like EfficientNet and Vision Transformers may restrict the investigation of possibly better models. Future study should focus on incorporating such architecture.

Additionally, compared to lightweight designs like MobileNetV2, performance gains come at the expense of higher computational complexity. When implementing the model in real-world clinical settings with constrained computational resources, this trade-off should be taken into account. All things considered, this study offers a clinically focused and methodically verified hybrid architecture that not only enhances classification performance but also tackles important diagnostic concerns, especially in lowering false-negative results.

Consequently, more recent architectures such as EfficientNet, RegNet, or Vision Transformers (ViT) were not included. While the selected models worked exceptionally well, exploring these novel architectures stays a major direction for future research to determine potentially higher-performing configurations. Comparative analysis with these models will provide a more comprehensive understanding of optimal architectures for breast cancer classification.

Lastly, moving forward, future studies will focus on the validation of larger, multi-center datasets and the testing of various hybrid integrations across different imaging protocols and patient populations. In addition, integration into clinical workflows will be explored to evaluate its potential as a real-time decision-support tool. To bridge the gap between AI performance and clinical trust, we also plan to combine explainable AI (XAI) methods, such as Grad-CAM. This will ensure visual interpretations of the model’s decision-making process, providing that the classification is dependent on relevant pathological characteristics rather than image artifacts.

## Data Availability

The dataset used in this study is publicly available on http://doi.org/10.7937/K9/TCIA.2015.SDNRQXXR.

## Abbreviations

Acc: Accuracy
AI: Artificial Intelligence
AUC: Area Under the Curve
CAD: Computer-Aided Diagnostic
CI: Confidence Intervals
CNN: Convolutional Neural Network
DCNN: Deep Convolutional Neural Network
DL: Deep Learning
FLOP: Floating Point Operations
FN: False Negative
FP: False Positive
FPR: False Positive Rate
FS: Feature Selection
GLOBOCAN: Global Cancer Observatory
GPU: Graphics Processing Unit
Grad-CAM: Gradient-weighted Class Activation Mapping
LSTM: Long-Short Term Memory
MRI: Magnetic Resonance Imaging
ms: Millisecond
RNN: Recurrent Neural Network
ROC: Receiver Operating Characteristic
SVM: Support Vector Machine
TL: Transfer Learning
TN: True Negative
TP: True Positive
TPR: True Positive Rate
ViT: Vision Transformers
XAI: Explainable Artificial Intelligence
WHO: World Health Organization
